# Enhanced Sensitivity of CO on Two-Dimensional, Strained, and Defective GaSe

**DOI:** 10.3390/molecules26040812

**Published:** 2021-02-04

**Authors:** Hsin-Pan Huang, Huei-Ru Fuh, Ching-Ray Chang

**Affiliations:** 1 Graduate Institute of Applied Physics, National Taiwan University, Taipei 10617, Taiwan; 2 Department of Chemical Engineering and Materials Science, Yuan Ze University, Taoyuan 32003, Taiwan; hrfuh@saturn.yzu.edu.tw; 3 Department of Physics, National Taiwan University, Taipei 10617, Taiwan; crchang@phys.ntu.edu.tw

**Keywords:** two-dimensional material, CO sensing, vacancy, strain effect, first-principles study

## Abstract

The toxic gas carbon monoxide (CO) is fatal to human beings and it is hard to detect because of its colorless and odorless properties. Fortunately, the high surface-to-volume ratio of the gas makes two-dimensional (2D) materials good candidates for gas sensing. This article investigates CO sensing efficiency with a two-dimensional monolayer of gallium selenide (GaSe) via the vacancy defect and strain effect. According to the computational results, defective GaSe structures with a Se vacancy have a better performance in CO sensing than pristine ones. Moreover, the adsorption energy gradually increases with the scale of tensile strain in defective structures. The largest adsorption energy reached −1.5 eV and the largest charger transfer was about −0.77 e. Additionally, the CO gas molecule was deeply dragged into the GaSe surface. We conclude that the vacancy defect and strain effect transfer GaSe to a relatively unstable state and, therefore, enhance CO sensitivity. The adsorption rate can be controlled by adjusting the strain scale. This significant discovery makes the monolayer form of GaSe a promising candidate in CO sensing. Furthermore, it reveals the possibility of the application of CO adsorption, transportation, and releasement.

## 1. Introduction

Two-dimensional (2D) layered materials have displayed a new stage ever since graphene was successfully fabricated in 2004 [1]. Recently, more and more 2D materials have been synthesized and investigated [2]. These include layered transition metal dichalcogenides (TMDs) ${\mathrm{AB}}_{2}$ (A = Mo, W, and Re and B = S and Se) and layered group III-VI semiconductors such as GaS and GaSe. The extraordinary physical and chemical properties for these 2D materials have drawn huge attention and have been widely studied [3,4]. One of the interesting properties of 2D materials is their high surface-to-volume ratio, which provides many reactive sites that are good candidates in gas sensing applications [5,6,7,8]. Further studies [9] have reported that some procedures, such as defect functionalization [10] (defects, doping, grain boundaries, and edge sites), heterojunction utilization [11], and electrical field application [12], can also improve gas sensing performance for 2D materials.

Since the industrial revolution, the global environment has been seriously polluted with the emission of carbon monoxide (CO). The capture and storage of these gas molecules is an important problem which needs to be solved as soon as possible. The toxic carbon monoxide gas can cause immediate death for humans, and it is colorless, tasteless, and hard to detect [13]. Therefore, finding proper materials for CO sensing is critically important. Early studies have shown that for metal-oxide semiconductor (MOS) sensors, their resistance changes with the environmental CO concentration [14,15]. If we can develop new devices to sense this toxic gas, this will benefit humans. Many 2D materials have been investigated in terms of their CO sensitivity, such as graphene [16,17], graphene oxide [18], silicene [19], ${\mathrm{WS}}_{2}$ [20], ${\mathrm{WSe}}_{2}$ [21], ${\mathrm{MoS}}_{2}$ [22,23], ${\mathrm{PtSe}}_{2}$ [24], and so on [25,26].

In experiments, the strain can be applied on 2D materials in several ways, such as deforming the substrate, creating wrinkles, using pre-patterned substrates, deforming a suspended membrane, lattice mismatch, and out-of-plane compression [27]. There are many interesting properties induced by the strain effect. The modulations were band structures [28], electrical properties [29], magnetic properties [30], optical properties [31], thermal conductivity [32], catalytic properties [33], phase transition and fracture [34], and interlayer coupling [35]. Therefore, the stain is regarded as a powerful tool cooperating with an investigation of 2D materials.

In this article, we use a monolayer of gallium selenide (GaSe) as a sensing material. GaSe is a III-VI semiconductor with extraordinary piezoelectric properties [36]. The electronic structure of GaSe has already been theoretically investigated [37,38]. For the monolayer structure, the energy gap decreases by applying a tensile strain [39]. In practical applications, because of the interesting properties of the material, such as continuous band gap tunability and photoluminescence enhancement, GaSe can be designed for use in wearable human devices [40]. An experimental study has also shown that GaSe can be fabricated as an ultrathin layer field-effect transistor [41]. Moreover, electrical resistance variation for two-dimensional wrinkle-based thin-film GaSe strain sensors is very sensitive to strain [42].

Recent research has indicated that biaxial strain does not significantly influence the adsorption stability of gas molecules on the GaSe monolayer [43]. Therefore, we combine the strain and defect effects to investigate how CO adsorbs on a monolayer GaSe. In this work, we calculate the variation of the electronic structure, differential charge density (DCD) patterns, adsorption energy, and charge transfer. According to the calculated results, the adsorption efficiencies of the Se-defected structures are better than those of the pristine ones. Furthermore, the biaxial tensile strain enhances the adsorption ability. By controlling the scale of tensile strain, we can change the adsorption rate. This discovery may be used in applications that deal with adsorbing, transporting, and releasing CO molecules.

## 2. Results

### 2.1. Pristine GaSe Crystal Structure

**Figure 1:**
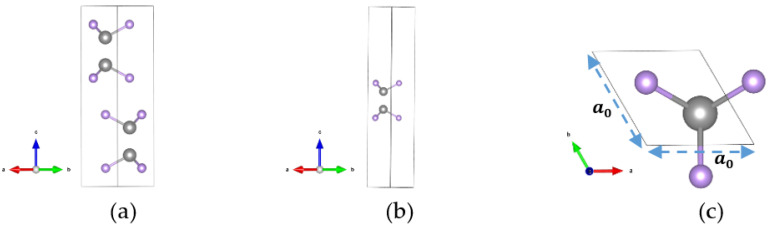
The crystal structure for the gallium selenide (GaSe) unit cell. (**a**) Side view of bulk. (**b**) Side view of 1 × 1 monolayer. (**c**) Top view of 1 × 1 monolayer. The colors gray and purple indicate Ga and Se atoms, respectively.

GaSe is a III-VI semiconductor which has many kinds of structures. In this study, we focus on the β-GaSe, which has a hexagonal structure and belongs to the P63/mmc group space [44]. For the bulk phase, the lattice constants of a unit cell are a=3.75 Å, b=3.75 Å, and c=16 Å, and the corresponding angles are $\alpha =90^{\circ },\;\beta =90^{\circ },\;\mathrm{and}\;\gamma =120^{\circ }$. The crystal structure of the bulk phase is shown in Figure 1a. The crystal structures of a monolayer GaSe unit cell, which contains two Ga atoms and two Se atoms, are shown in Figure 1b,c. In the following section, we focus on different sizes of monolayer GaSe.

### 2.2. The Structure and Adsorption Energy of CO Adsorbed on a Monolayer GaSe Supercell

**Figure 2:**
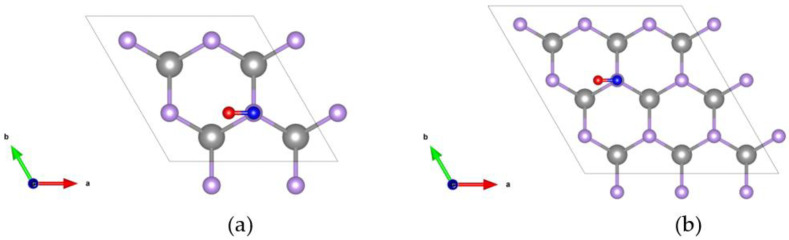
Top view of the crystal structure of a CO gas molecule adsorped on the monolayer GaSe supercell. (**a**) 2 × 2 (**b**) and 3 × 3 supercells. The colors for Ga, Se, C, and O are gray, purple, blue, and red, respectively.

To investigate CO adsorption with a monolayer GaSe, we enlarged the unit cell into 2 × 2 and 3 × 3 supercells, with one CO gas molecule on each surface. This allows us to compare the adsorption efficiencies between different CO concentrations. The corresponding crystal structures of the 2 × 2 and 3 × 3 supercells are shown in Figure 2a,b, respectively. The Se atom on top of the monolayer GaSe is removed when considering the defect effect.

#### 2.2.1. Crystal Structure of CO Adsorbed on the GaSe Monolayer

**Figure 3:**
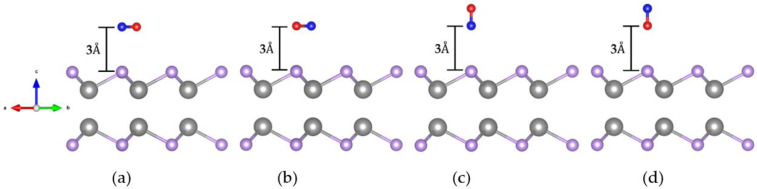
Crystal structures for CO adsorption on a 2 × 2 monolayer GaSe supercell. (**a**) Horizontal orientation with a C atom above the Se stom. (**b**) Horizontal orientation with an O atom above the Se atom. (**c**) Vertical orientation with a C atom below and an O atom above. (**d**) Vertical orientation with an O atom below and a C atom above.

Firstly, we chose the Se atom on the top of the GaSe surface as the site where the CO molecule absorbed. To find the most favorable configuration, we investigated four different initial orientations of the CO gas molecule as adsorbed on the monolayer GaSe. The crystal structures, with the two in the horizontal direction and the two in vertical direction, are shown in Figure 3a–d, respectively. The initial vertical distance between the CO molecule and the top Se atom in the GaSe surface is 3 Å.

The calculation results are listed in Table 1. The pristine structures with initial orientation A, B, C, and D are shown in Figure 3a–d, respectively. The adsorption parameters are described as follows: $E_{rel}$ is the relative energy, $E_{ad}$ is the adsorption energy, ΔQ is charge transfer, and *h* is the final vertical distance between CO and the GaSe surface. *d*_1_ is the final distance between the lower atom in the CO molecule and the nearest Se atom in the top GaSe surface. When considering a Se vacancy, the final distance between the lower atom in CO and the nearest Ga atom is noted as *d*_2_. As a reference, we set the lowest total energy of the GaSe-CO system to zero. The relative energy $E_{rel}$ denotes this lowest total energy was subtracted from the higher total energies.

For both the pristine and defective cases, the total energies of the initial horizontal orientations were lower than the vertical ones. The total energies of two horizontal orientations, A and B, were almost the same, with only a 10 meV difference. Moreover, the $E_{ad}$ values indicate that the horizontal orientations were more sensitive than the vertical ones, no matter whether considering pristine or Se-defective GaSe. Therefore, in the following discussion, we emphasize the horizontal orientation A for both pristine and Se-defective GaSe.

#### 2.2.2. CO Adsorption without Strain Effect

In order to discuss how different CO concentrations adsorb on monolayer GaSe, we constructed 2 × 2 and 3 × 3 supercells. The adsorption parameters for CO adsorption on the monolayer GaSe are listed in Appendix C, Table A1. For the pristine 2 × 2 supercell, the vertical distance was enlarged to 3.5 Å after relaxation. The adsorption energy of CO adsorbed on GaSe was −64.89 meV. The charge transfer was −0.017 e. The negative sign represents charge transfer from GaSe to the CO molecule. For the defective 2 × 2 supercell, the CO molecule was attracted toward GaSe surface with a final vertical distance of 0.37 Å. The adsorption energy was −238.49 meV and the amount of charge transfer was −0.143 e. Both the adsorption energy and charge transfer in the defective structure were far larger than in the pristine structure.

The behaviors for the 3 × 3 supercell were similar to those for the 2 × 2 supercell. For the pristine 3 × 3 supercell, the vertical distance was 3.53 Å. The adsorption energy was −65.59 meV and the amount of charge transfer was −0.015 e. For the defective 3 × 3 supercell, the adsorption energy was −146.07 meV and the amount of charge transfer was −0.055 e. After the relaxation process, the CO molecule approached the GaSe surface and the final vertical distance was 1.93 Å. Both the increase in the adsorption energy and charge transfer were weaker than that with the 2 × 2 supercell, which can be explained by the lower concentration of Se vacancy in the 3 × 3 supercell.

#### 2.2.3. CO Adsorption with Strain Effect

For the pristine structures, the strain effect did not influence the CO sensitivity in both the 2 × 2 and 3 × 3 supercells; however, the adsorption energies were obviously enhanced in the defective structure. When considering the strain effect, we modulated the scaling factor of a reciprocal lattice vector in each crystal structure file. The original scaling factor without strain effect was 1.0, while 1% tensile strain was denoted as 1.01. According to Table A1, while the tensile strain gradually increased from 1% to 5%, the adsorption energy correspondingly enhanced from −428.67 meV to −844.39 meV in the 2 × 2 supercell and from −141.65 meV to −1515.68 meV in the 3 × 3 supercell.

**Figure 4:**
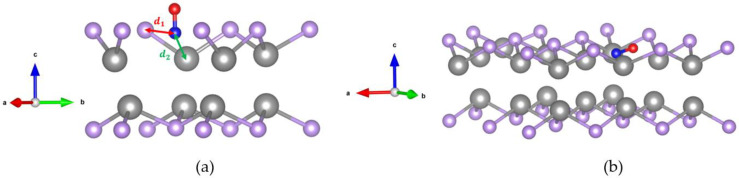
The relaxation structure of CO adsorbed on defective GaSe while considering +3% tensile strain. (**a**) 2 × 2 (**b**) and 3 × 3 supercells.

The relaxation structures of CO adsorption in the 2 × 2 and 3 × 3 defective GaSe supercells are shown in Figure 4a,b, respectively. We applied the strain effect on both supercells from −5% to 5%. A mid-value tensile strain scale of +3% is chosen as an example. For the 2 × 2 supercell, the CO molecule reaches a vertical C-down and O-up configuration, as shown in Figure 4a. For 3 × 3 dimensions, the CO molecule tilts with the C atom slightly lower than O atom, as shown in Figure 4b. In Figure 4a, $d_{1}$ is the nearest distance between a C atom and Se atom, while $d_{2}$ is the nearest distance between a C atom and a Ga atom. As CO molecules are deeply attracted into GaSe in both cases, the C atom is closer to the Ga atom rather than the Se atom. Therefore, the $d_{2}$ value is smaller than $d_{1}$.

### 2.3. Electronic Structure Analysis

#### 2.3.1. Pristine GaSe

The intrinsic properties of GaSe have already been systematically studied in earlier research [39]. Our calculations show similar results. Thus, the calculation results are only briefly mentioned and the details are provided in Appendix A.

For bulk GaSe, the energy gap is 1.01 eV with a direct band gap. This result was smaller than the experimental data, with about 2.1 eV [45]. It is not surprising to see the underestimation of the band gaps because of the restriction of the generalized gradient approximation (GGA) method [46]. For the monolayer, the result shows an indirect band gap with a 1.79 eV energy gap.

In previous theoretical study, the energy gap of GaSe has been systematically investigated from bulk, four-layer, bilayer, to monolayer forms [39]. The direct gap is about 0.995 eV for the bulk form and the indirect gap is about 2.252 eV for the monolayer form.

#### 2.3.2. Pristine Monolayer GaSe with Strain Effect

We investigated the biaxial strain effect with a monolayer unit cell, considering both compressive and tensile strain. The strain effect was achieved by adjusting the scaling factor of a reciprocal lattice vector. The original scaling factor was 1.0, larger than 1.0 for tensile strain, and lower than 1.0 for compressive strain. The scaling factor of 1.01 was defined as 1% tensile strain, while 0.99 corresponded to −1% compressive strain. The scale of strain in our study ranges from −5% to 5%.

**Figure A2:**
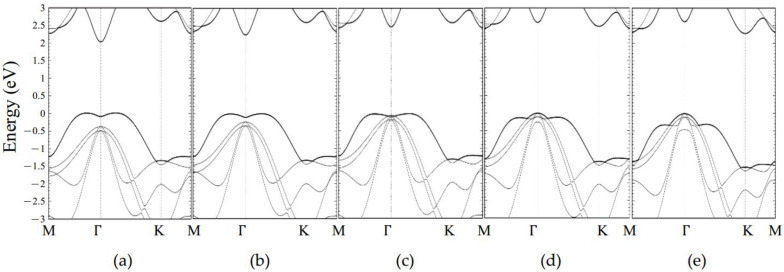
The electronic structure for a 1 × 1 monolayer of GaSe when applying compressive strain. (**a**) −1%; (**b**) −2%; (**c**) −3%; (**d**) −4%; (**e**) −5%.

**Figure A3:**
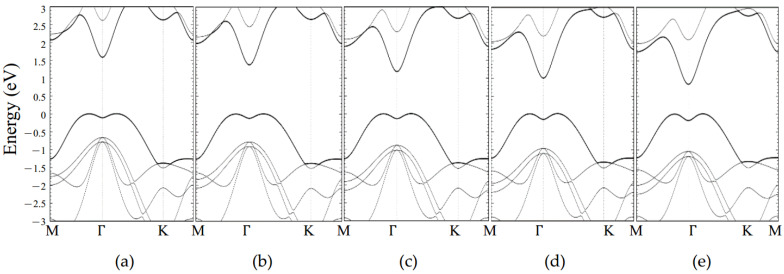
The electronic structure for a 1 × 1 monolayer of GaSe when applying tensile strain. (**a**) 1%; (**b**) 2%; (**c**) 3%; (**d**) 4%; (**e**) 5%.

The calculation results for the corresponding energy gaps are listed in Table A1. The energy gap decreased with an increase in tensile strain, while raising with an increase in compressive strain. This behavior is consistent with previous studies [39,47]. The reason for the slight decrease in the energy gap for −4% and −5% compressive strain is the change of the conduction band minimum. Detailed descriptions of the electronic structures are shown in Appendix B in Figure A2 and Figure A3.

#### 2.3.3. CO Adsorption with Monolayer GaSe

**Figure 5:**
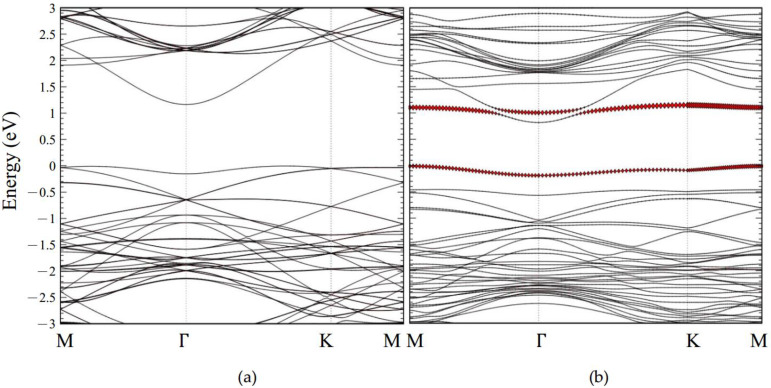
The electronic structures of 3 × 3 monolayer GaSe adsorbed with a CO molecule with a tensile strain of 3% and initial orientation A. (**a**) Pristine structure. (**b**) Defective structure.

The electronic structures of CO adsorption on 3 × 3 pristine and defective GaSe supercells with 3% tensile strain are shown in Figure 5a,b, respectively. The Fermi level has been shifted to 0 eV and the red dots demonstrate the occupation of electrons contributed by the CO molecule. For CO the molecule adsorption on pristine GaSe in Figure 5a, the energy gap is 1.17 eV. The electron occupation is barely contributed by the CO molecule, which is in the energy range of −3 eV to 3 eV; however, for a CO molecule adsorbing on the defective GaSe, the CO molecule decomposes the band structure and has an obvious distribution near the Fermi level, as shown in Figure 5b. This special phenomenon has been caused by the CO molecule being tightly trapped in the defective monolayer of GaSe. In addition, this would induce dramatic variation in charge transfer and adsorption energy values. 

### 2.4. Differential Charge Densities Pattern Analysis

To demonstrate the charge accumulation/depletion between a CO gas molecule and monolayer GaSe, we calculated differential charge densities (DCDs) by using the following equation [48]:

(1) $$\rho_{DCDs}=\rho_{system}-\left(\rho_{GaSe}+\rho_{CO}\right),$$

where $\rho_{system}$ is the charge density for the system of the monolayer GaSe when adsorbing a CO gas molecule, while $\rho_{GaSe}$ and $\rho_{CO}$ correspondingly represent charge densities of monolayer GaSe and the individual CO molecule.

**Figure 6:**
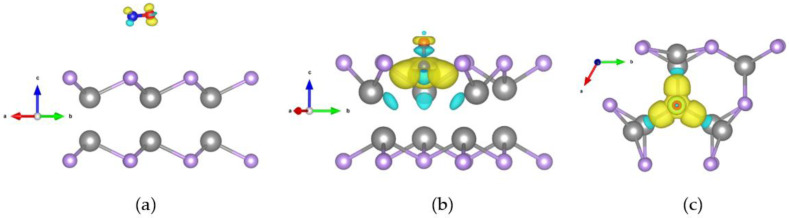
Differential charge density pattern with a 2 × 2 monolayer GaSe adsorbed with a CO molecule while considering a 3% tensile strain. The isosurface is $0.0005\;e/bohr^{3}$. (**a**) Pristine structure; (**b**) side view of defective structure (**c**); top view of defective structure.

**Figure 7:**
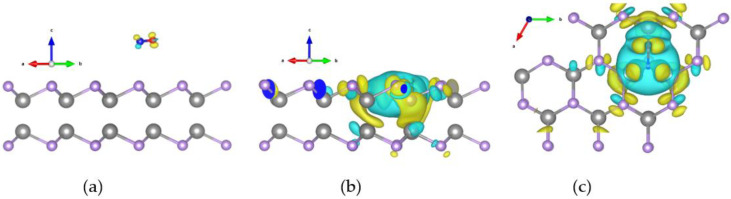
Differential charge density patterns of 3 × 3 monolayer GaSe adsorbed with a CO molecule, while considering a 3% tensile strain. The isosurface is $0.0005\;e/bohr^{3}$. (**a**) Pristine structure; (**b**) side view of defective structure; (**c**) top view of defective structure.

The DCD patterns of CO adsorption on the pristine and defective GaSe, corresponding to the 2 × 2 and 3 × 3 supercells, correspond to Figure 6 and Figure 7, respectively. We demonstrate a 3% tensile strain in both figures. In Figure 6 and Figure 7, section (a) indicates a pristine GaSe structure, while sections (b) and (c) represent side and top views of a defective structure, respectively. The isosurfaces are all set as $0.0005\;e/bohr^{3}$ for consistency. The electron accumulation is demonstrated by a yellow color, while the light blue color represents charge depletion.

For the pristine structures, the charge transfer amounts were −0.014 e for the 2 × 2 supercell and −0.017e for the 3 × 3 supercell. Both values are very small, as shown in Figure 6a and Figure 7a. For the defective structure, the amount of charge transfer was −0.096 e for the 2 × 2 supercell and −0.776 e for the 3 × 3 supercell. Both values are very large when compared with the pristine GaSe, as shown in Figure 6b,c and Figure 7b,c.

In the DCD patterns, the regions of charge accumulation and depletion were significantly different between the pristine and defective forms. This phenomenon arises due to the enhancement of charge transfer, which also provides a good explanation for the improvement of the sensing ability. It is easy to comprehend that for a defective structure, the arrangement of the atoms is destroyed, and this results in a nonuniform distribution of electrons. This unstable system forms a net force, which pulls the CO molecules toward the GaSe surface and then finally reaches a new equilibrium situation.

## 3. Discussion

**Figure 8:**
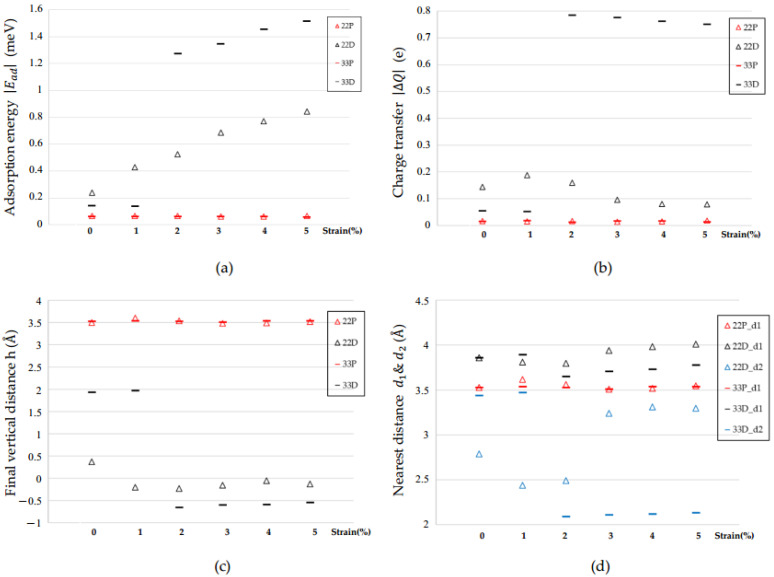
The comparison patterns of the adsorption parameters (initial orientation A). (**a**) Adsorption energy $\left|E_{ad}\right|$; (**b**) charge transfer |ΔQ|; (**c**) final vertical distance *h*; (**d**) nearest distances $d_{1}$ and $d_{2}$.

The calculation results of $\left|E_{ad}\right|$, |ΔQ|, *h*, $d_{1}$, and $d_{2}$ are shown in Figure 8a–d. The notations of 22 and 33 represent the 2 × 2 and 3 × 3 supercells, which are symbolized as triangles and bars, respectively. The notations P and D represent the pristine and defective structure shown in red and black, respectively.

In Figure 8a, the magnitudes of the adsorption energy $\left|E_{ad}\right|$ for pristine structures are very small and are in the range of 64.54 meV to 61.31 meV from 1% to 5% strain for a 3 × 3 supercell, respectively. When considering Se defect vacancy, there is a significant increase in the range of 141.65 meV to 1515.68 meV from 1% to 5% strain for a 3 × 3 supercell, respectively. While applying tensile strain of +5%, the magnitudes of the adsorption energy exceed 0.8 eV in the 2 × 2 supercell and 1.5 eV in the 3 × 3 supercell.

In Figure 8b, the magnitudes of charge transfer |ΔQ| for pristine structures are also very small and are in the range of 0.017 e to 0.014 e from 1% to 5% strain for a 3 × 3 supercell, respectively. While considering both the vacancy and strain effect, the values are in the range of 0.052 e to 0.75 e from 1% to 5% strain for a 3 × 3 supercell. The magnitudes of charge transfer approach about 0.2 e in a 2 × 2 supercell and 0.8 e in a 3 × 3 supercell with defect and strain effect.

In Figure 8c, the final vertical distance for the pristine structure is about 3.5 Å, which is farther than the initial distance of 3 Å. While considering the defect and strain effects, the CO molecules are attracted toward the GaSe surface. Therefore, the final vertical distances are smaller than 3 Å and even become negative with strain effect. The negative value represents that the C atom of the CO molecule is lower than the top Se atom in the GaSe surface.

In Figure 8d, the values of nearest distance between the CO and GaSe surfaces in the pristine structure are similar to the vertical distance (the red symbols). For the defective structure, because CO molecules are attracted toward the GaSe surface, the CO molecules are closer to the Ga atom than the Se atom. Therefore, the $d_{2}$ values (the blue symbols) are smaller than the $d_{1}$ values (the black symbols). 

## 4. Materials and Methods

This study was performed with first-principles calculations based on density functional theory (DFT), which were performed via a pseudopotential method. A projector-augmented-wave (PAW) was employed and the calculation was implemented in the Vienna ab initio simulation package (VASP) [49]. For the exchange-correlation term in the Kohn–Sham equation, the generalized gradient approximation (GGA) [50] in the Perdew–Burke–Ernzerhof (PBE) scheme [51] was used. The van der Waals force calculations were evaluated with the DFT-D2 correction of Grimme [52]. The k-point meshes were set as 13 × 13 × 3 for the bulk phase and 10 × 10 × 1 for the monolayer. The cutoff energy was set to 400 eV for the plane-wave expansion. The energy convergence criterion was set $10^{-5}$ eV to ensure the accuracy in the self-consistent process. The force convergence tolerance for each atom was less than 0.02 eV/Å.

The crystal structures and differential charge density patterns were demonstrated by visualization for electronic and structural analysis (VESTA) [53]. To avoid interaction, the vacuum regions between adjacent slabs were set to about 20 Å along the *Z*-axis. The charge transfer values between the monolayer GaSe and CO gas molecule were calculated via Bader charge analysis [54,55].

The adsorption energy ($E_{ad}$) was calculated with following equation [56,57]:

(2) $$E_{ad}=E_{system}-\left(E_{GaSe}+E_{CO}\right),$$

where $E_{system}$ is the total energy for the system after CO is adsorbed on the monolayer GaSe. $E_{GaSe}$ and $E_{CO}$ correspond to the total energies of the monolayer GaSe and the CO gas molecule before adsorption. The negative value of the adsorption energy reveals that gas adsorption may spontaneously occur.

## 5. Conclusions

A defective monolayer of GaSe with Se vacancy has better sensitivity than a pristine structure, where both the adsorption energy and charge transfer are significantly enhanced. The adsorption energies of a CO molecule adsorbed on pristine and defective monolayers of GaSe here ranged from −64.89 meV to −238.49 meV in the 2 × 2 supercell and from −65.59 meV to −146.07 meV in the 3 × 3 supercell, respectively. The charge transfer increased from −0.017 e to −0.143 e in the 2 × 2 supercell and from −0.015 e to −0.055 e in the 3 × 3 supercell.

In addition, when considering the strain effect with Se vacancy, the adsorption energies gradually increased with the strain scales. The adsorption energies raised from −428.67 meV to −844.39 meV in the 2 × 2 supercell and from −141.07 meV to −1515.68 meV in the 3 × 3 supercell. This behavior can be inferred from the imperfect structure, which causes the redistribution of electrons and induces an attractive force on the CO molecules. Evidence is given by the relaxation structure where CO molecules are pulled toward the GaSe surface in the final position.

Both electronic structure and differential charge density patterns show obvious variation with the defective condition. For electronic structures, the emergence of a flat band near the Fermi level and the redistribution of electron occupation illustrate the enhancement of the charge transfer amount. For differential charge density patterns, the extension of the charge accumulation and depletion area indicate the enhancement of the interaction between the CO molecule and defective monolayer of GaSe.

According to the investigation results, the CO sensitivity in defective monolayer GaSe structure can be affected by the scale of the tensile strain. A previous study has revealed that the strain effect can be fulfilled in two-dimensional wrinkle-based GaSe [40]. At present, it is also possible to fabricate a defective material via experimental technology. As the adsorption ability can be tuned by the scale of strain, GaSe can be regarded as a potential candidate to capture, transport, and release CO gas molecules.

## Figures and Tables

**Table 1:** The comparison of different initial orientations of CO adsorbed on a 2 × 2 GaSe supercell.

GaSe	Orientation	$E_{rel}\;\left(\mathrm{eV}\right)$	$E_{ad}\;(\mathrm{meV})$	ΔQ (e)	h (Å)	$d_{1}\;(Å)$	$d_{2}\;(Å)$
Pristine	A	0.01	−64.89	−0.017	3.50 (C-Se) ^1^	3.53 (C-Se)	-
	B	0	−70.13	−0.013	3.13 (C-Se)	3.54 (C-Se)	-
	C	0.04	−33.85	−0.005	3.85 (C-Se)	3.85 (C-Se)	-
	D	0.03	−36.42	−0.007	3.66 (O-Se)	3.67 (O-Se)	-
Se-defective	A	0	−238.49	−0.143	0.37 (C-Se)	3.86 (C-Se)	2.79 (C-Ga)
	B	0.01	−321.60	−0.119	0.58 (C-Se)	3.92 (C-Se)	3.93 (C-Ga)
	C	0.24	−91.38	−0.012	2.54 (C-Se)	4.59 (C-Se)	4.19 (C-Ga)
	D	0.25	−79.81	−0.015	2.39 (O-Se)	4.50 (O-Se)	4.05 (O-Ga)

(C-Se) ^1^ represents the distance measured between a C atom and a Se atom.

**Figure A1:**
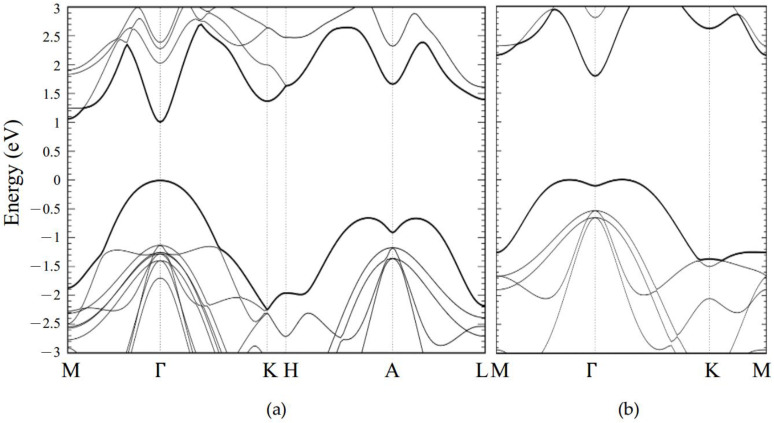
Electronic structure for (**a**) a bulk phase (**b**) and a 1 × 1 monolayer unit cell.

## Data Availability

Not applicable.

## Appendix A. Electronic Structure for Pristine GaSe

The electronic structures of GaSe are shown in Figure A1, where (a) and (b) represent a bulk and 1 × 1 monolayer unit cell. For bulk GaSe, the van der Waals force with the DFT-D2 method was considered. The calculation results demonstrate a direct energy gap of 1.01 eV for the bulk state and an indirect band gap value of 1.79 eV for the 1 × 1 monolayer configuration. A detailed description of the calculation is included in the text.

## Appendix B. Electronic Structure for Monolayer GaSe with Strain Effect

The definition of the strain scale has been mentioned in the text and the values of the energy gaps are listed in Table A1. The electronics structures for the 1 × 1 monolayer unit cell of GaSe are shown in Figure A2 and Figure A3, corresponding to compressive and tensile strain. The energy gaps increase with the increase in compressive strain and decrease with the increase in tensile strain.

**Table A1:** Energy gap for monolayer GaSe with the strain effect.

Strain	−5%	−4%	−3%	−2%	−1%	0%	1%	2%	3%	4%	5%
$E_{g}\left(\mathrm{eV}\right)$	2.29	2.43	2.45	2.24	2.02	1.79	1.58	1.37	1.18	1.00	0.83

## Appendix C. Calculation Results of Monolayer GaSe Adsorbed with CO

Table A1 lists the parameters for CO adsorption on GaSe supercells (initial orientation A). In this table, 2 × 2 and 3 × 3 supercells are considered with pristine and defective structure, both with and without strain effects. The parameters are defined in the main text. $E_{rel}$ is the relative energy. The total energy of the GaSe-CO system without adding strain is set to zero in each case. The relative energy denotes the total energy without strain subtracted from total energy with strain.

**Table A2:** Parameters of CO adsorption on a monolayer of GaSe.

Supercell	GaSe Structure	Strain (%)	$E_{rel}\;(\mathrm{eV})$	$E_{ad}\;(\mathrm{meV})$	ΔQ (e)	h (Å)	$d_{1}\;(Å)$	$d_{2}\;(Å)$
2 × 2	Pristine	0	0	−64.89	−0.017	3.50	3.53	-
		1	0.13	−64.79	−0.015	3.60	3.62	-
		2	0.32	−64.41	−0.016	3.54	3.56	-
		3	0.55	−62.64	−0.014	3.48	3.51	-
		4	0.82	−62.00	−0.015	3.49	3.52	-
		5	1.13	−63.72	−0.018	3.52	3.55	-
	Se-defective	0	0	−238.49	−0.143	0.37	3.86	2.79
		1	0.1	−428.67	−0.188	−0.20	3.81	2.44
		2	0.24	−524.80	−0.160	−0.23	3.80	2.49
		3	0.35	−684.13	−0.096	−0.16	3.94	3.24
		4	0.53	−771.41	−0.081	−0.06	3.98	3.31
		5	0.74	−844.39	−0.079	−0.13	4.01	3.30
3 × 3	Pristine	0	0	−65.59	−0.015	3.53	3.53	-
		1	0.32	−64.54	−0.017	3.54	3.54	-
		2	0.74	−63.58	−0.013	3.53	3.53	-
		3	1.26	−62.56	−0.017	3.51	3.51	-
		4	1.87	−62.64	−0.017	3.54	3.54	-
		5	2.55	−61.31	−0.014	3.54	3.54	-
	Se-defective	0	0	−146.07	−0.055	1.93	3.86	3.44
		1	0.4	−141.65	−0.052	1.97	3.89	3.47
		2	-0.25	−1275.20	−0.784	−0.66	3.65	2.09
		3	0.22	−1374.28	−0.776	−0.60	3.71	2.11
		4	0.78	−1456.81	−0.763	−0.59	3.73	2.12
		5	1.42	−1515.68	−0.750	−0.55	3.78	2.13

^1^ (C-Se) represents the distance between a C atom and a Se atom.

## References

[1] Novoselov K.S., Geim A.K., Morozov S.V., Jiang D., Zhang Y., Dubonos S.V., Grigorieva I.V., Firsov A.A. (2004). Electric field effect in atomically thin carbon films. Science.

[2] Druffel D.L., Woomer A.H., Kuntz K.L., Pawlik J.T., Warren S.C. (2017). Electrons on the surface of 2D materials: From layered electrides to 2D electrenes. J. Mater. Chem. C.

[3] Wang X., Sun Y., Liu K. (2019). Chemical and structural stability of 2D layered materials. 2D Mater..

[4] Zhang C.-W. (2012). First-principles study on electronic structures and magnetic properties of AlN nanosheets and nanoribbons. J. Appl. Phys..

[5] Yang S., Jiang C., Wei S.-H. (2017). Gas sensing in 2D materials. Appl. Phys. Rev..

[6] Liu X., Ma T., Pinna N., Zhang J. (2017). Two-Dimensional Nanostructured Materials for Gas Sensing. Adv. Funct. Mater..

[7] Varghese S., Varghese S., Swaminathan S., Singh K., Mittal V. (2015). Two-Dimensional Materials for Sensing: Graphene and Beyond. Electronics.

[8] Ouyang T., Qian Z., Hao X., Ahuja R., Liu X. (2018). Effect of defects on adsorption characteristics of AlN monolayer towards SO_2_ and NO_2_: Ab initio exposure. Appl. Surf. Sci..

[9] Zeng Y., Lin S., Gu D., Li X. (2018). Two-Dimensional Nanomaterials for Gas Sensing Applications: The Role of Theoretical Calculations. Nanomaterials.

[10] Zhang Y.H., Chen Y.B., Zhou K.G., Liu C.H., Zeng J., Zhang H.L., Peng Y. (2009). Improving gas sensing properties of graphene by introducing dopants and defects: A first-principles study. Nanotechnology.

[11] Sun J., Lin N., Ren H., Tang C., Yang L., Zhao X. (2016). Gas adsorption on MoS_2_/WS_2_ in-plane heterojunctions and the I–V response: A first principles study. RSC Adv..

[12] Ao Z.M., Li S., Jiang Q. (2010). Correlation of the applied electrical field and CO adsorption/desorption behavior on Al-doped graphene. Solid State Commun..

[13] Raub J.A., Mathieu-Nolf M., Hampson N.B., Thom S.R. (2000). Carbon monoxide poisoning—A public health perspective. Toxicology.

[14] Nandy T., Coutu R.A., Ababei C. (2018). Carbon Monoxide Sensing Technologies for Next-Generation Cyber-Physical Systems. Sensors.

[15] Yamazoe N., Sakai G., Shimanoe K. (2003). Oxide semiconductor gas sensors. Catal. Surv. Asia.

[16] Leenaerts O., Partoens B., Peeters F.M. (2008). Adsorption of H_2_O, NH_3_, CO, NO_2_, and NO on graphene: A first-principles study. Phys. Rev. B.

[17] Kumar J., Nemade H.B., Giri P.K. (2018). Adsorption of Small Molecules on Niobium Doped Graphene: A Study Based on Density Functional Theory. IEEE Electron. Device Lett..

[18] Wang L., Zhao J., Wang L., Yan T., Sun Y.-Y., Zhang S.B. (2011). Titanium-decorated graphene oxide for carbon monoxide capture and separation. Phys. Chem. Chem. Phys..

[19] Zhong S., Ning F., Rao F., Lei X., Wu M., Zhou L. (2016). First-principles study of nitrogen and carbon monoxide adsorptions on silicene. Int. J. Mod. Phys. B.

[20] Bui V.Q., Pham T.T., Le D.A., Thi C.M., Le H.M. (2015). A first-principles investigation of various gas (CO, H_2_O, NO, and O_2_) absorptions on a WS_2_ monolayer: Stability and electronic properties. J. Phys. Condens. Matter.

[21] Wang T., Zhao R., Zhao X., An Y., Dai X., Xia C. (2016). Tunable donor and acceptor impurity states in a WSe_2_ monolayer by adsorption of common gas molecules. RSC Adv..

[22] Ma D., Ju W., Li T., Zhang X., He C., Ma B., Lu Z., Yang Z. (2016). The adsorption of CO and NO on the MoS_2_ monolayer doped with Au, Pt, Pd, or Ni: A first-principles study. Appl. Surf. Sci..

[23] Fan Y., Zhang J., Qiu Y., Zhu J., Zhang Y., Hu G. (2017). A DFT study of transition metal (Fe, Co, Ni, Cu, Ag, Au, Rh, Pd, Pt and Ir)-embedded monolayer MoS2 for gas adsorption. Comput. Mater. Sci..

[24] Zhang J., Yang G., Tian J., Ma D., Wang Y. (2018). First-principles study on the gas sensing property of the Ge, As, and Br doped PtSe_2_. Mater. Res. Express.

[25] Cheng W.-Y., Fuh H.-R., Chang C.-R. (2020). First-Principles Study for Gas Sensing of Defective SnSe_2_ Monolayers. Appl. Sci..

[26] Beheshtian J., Baei M.T., Peyghan A.A. (2012). Theoretical study of CO adsorption on the surface of BN, AlN, BP and AlP nanotubes. Surf. Sci..

[27] Sun Y., Liu K. (2019). Strain engineering in functional 2-dimensional materials. J. Appl. Phys..

[28] Conley H.J., Wang B., Ziegler J.I., Haglund R.F., Pantelides S.T., Bolotin K.I. (2013). Bandgap engineering of strained monolayer and bilayer MoS_2_. Nano Lett..

[29] Fei R., Yang L. (2014). Strain-engineering the anisotropic electrical conductance of few-layer black phosphorus. Nano Lett..

[30] Guinea F., Katsnelson M.I., Geim A.K. (2009). Energy gaps and a zero-field quantum Hall effect in graphene by strain engineering. Nat. Phys..

[31] Li H., Contryman A.W., Qian X., Ardakani S.M., Gong Y., Wang X., Weisse J.M., Lee C.H., Zhao J., Ajayan P.M. (2015). Optoelectronic crystal of artificial atoms in strain-textured molybdenum disulphide. Nat. Commut..

[32] Kuang Y., Lindsay L., Huang B. (2015). Unusual Enhancement in Intrinsic Thermal Conductivity of Multilayer Graphene by Tensile Strains. Nano Lett..

[33] Li H., Tsai C., Koh A.L., Cai L., Contryman A.W., Fragapane A.H., Zhao J., Han H.S., Manoharan H.C., Abild-Pedersen F. (2016). Activating and optimizing MoS_2_ basal planes for hydrogen evolution through the formation of strained sulphur vacancies. Nat. Mater..

[34] Apte A., Kochat V., Rajak P., Krishnamoorthy A., Manimunda P., Hachtel J.A., Idrobo J.C., Syed Amanulla S.A., Vashishta P., Nakano A. (2018). Structural Phase Transformation in Strained Monolayer MoWSe_2_ Alloy. ACS Nano.

[35] Pak S., Lee J., Lee Y.W., Jang A.R., Ahn S., Ma K.Y., Cho Y., Hong J., Lee S., Jeong H.Y. (2017). Strain-Mediated Interlayer Coupling Effects on the Excitonic Behaviors in an Epitaxially Grown MoS_2_/WS_2_ van der Waals Heterobilayer. Nano Lett..

[36] Li W., Li J. (2015). Piezoelectricity in two-dimensional group-III monochalcogenides. Nano Res..

[37] Plucinski L., Johnson R.L., Kowalski B.J., Kopalko K., Orlowski B.A., Kovalyuk Z.D., Lashkarev G.V. (2003). Electronic band structure of GaSe(0001): Angle-resolved photoemission andab initiotheory. Phys. Rev. B.

[38] Rybkovskiy D.V., Arutyunyan N.R., Orekhov A.S., Gromchenko I.A., Vorobiev I.V., Osadchy A.V., Salaev E.Y., Baykara T.K., Allakhverdiev K.R., Obraztsova E.D. (2011). Size-induced effects in gallium selenide electronic structure: The influence of interlayer interactions. Phys. Rev. B.

[39] Ma Y., Dai Y., Guo M., Yu L., Huang B. (2013). Tunable electronic and dielectric behavior of GaS and GaSe monolayers. Phys. Chem. Chem. Phys..

[40] Wu Y., Fuh H.-R., Zhang D., Coileáin C.Ó., Xu H., Cho J., Choi M., Chun B.S., Jiang X., Abid M. (2017). Simultaneous large continuous band gap tunability and photoluminescence enhancement in GaSe nanosheets via elastic strain engineering. Nano Energy.

[41] Late D.J., Liu B., Luo J., Yan A., Matte H.S., Grayson M., Rao C.N., Dravid V.P. (2012). GaS and GaSe ultrathin layer transistors. Adv. Mater..

[42] Wang C., Yang S.-X., Zhang H.-R., Du L.-N., Wang L., Yang F.-Y., Zhang X.-Z., Liu Q. (2016). Synthesis of atomically thin GaSe wrinkles for strain sensors. Front. Phys..

[43] Zhou C., Zhu H., Wu Y., Lin W., Yang W., Dong L. (2017). Effect of external strain on the charge transfer: Adsorption of gas molecules on monolayer GaSe. Mater. Chem. Phys..

[44] Ueno K., Takeda N., Sasaki K., Koma A. (1997). Investigation of the growth mechanism of layered semiconductor GaSe. Appl. Surf. Sci..

[45] Fan Y., Schittkowski T., Bauer M., Kador L., Allakhverdiev K.R., Salaev E.Y. (2002). Confocal photoluminescence studies on GaSe single crystals. J. Lumin..

[46] Perdew J.P., Yang W., Burke K., Yang Z., Gross E.K., Scheffler M., Scuseria G.E., Henderson T.M., Zhang I.Y., Ruzsinszky A. (2017). Understanding band gaps of solids in generalized Kohn-Sham theory. Proc. Natl. Acad. Sci. USA.

[47] Yagmurcukardes M., Senger R.T., Peeters F.M., Sahin H. (2016). Mechanical properties of monolayer GaS and GaSe crystals. Phys. Rev. B.

[48] Camargo Moreira O.L., Cheng W.Y., Fuh H.R., Chien W.C., Yan W., Fei H., Xu H., Zhang D., Chen Y., Zhao Y. (2019). High Selectivity Gas Sensing and Charge Transfer of SnSe_2_. ACS Sens..

[49] Kresse G., Hafner J. (1993). Ab initio molecular dynamics for liquid metals. Phys. Rev. B Condens. Matter.

[50] Perdew J.P., Burke K., Ernzerhof M. (1996). Generalized gradient approximation made simple. Phys. Rev. Lett..

[51] Blochl P.E. (1994). Projector augmented-wave method. Phys. Rev. B Condens. Matter.

[52] Grimme S. (2006). Semiempirical GGA-type density functional constructed with a long-range dispersion correction. J. Comput. Chem..

[53] Momma K., Izumi F. (2011). VESTA 3 for three-dimensional visualization of crystal, volumetric and morphology data. J. Appl. Crystallogr..

[54] Henkelman G., Arnaldsson A., Jónsson H. (2006). A fast and robust algorithm for Bader decomposition of charge density. Comput. Mater. Sci..

[55] Sanville E., Kenny S.D., Smith R., Henkelman G. (2007). Improved grid-based algorithm for Bader charge allocation. J. Comput. Chem..

[56] Zhao S., Xue J., Kang W. (2014). Gas adsorption on MoS_2_ monolayer from first-principles calculations. Chem. Phys. Lett..

[57] Jiao Y., Zheng Y., Smith S.C., Du A., Zhu Z. (2014). Electrocatalytically switchable CO_2_ capture: First principle computational exploration of carbon nanotubes with pyridinic nitrogen. ChemSusChem.

